# 21.3 NEUROIMAGING MARKERS OF RISK FOR AND PROGRESSION TO FULL PSYCHOSIS IN THE NAPLS PROJECT

**DOI:** 10.1093/schbul/sby014.087

**Published:** 2018-04-01

**Authors:** Tyrone Cannon

**Affiliations:** Yale University

## Abstract

**Background:**

Dr. Larry Seidman made numerous impactful contributions to our understanding of the roles of disrupted neurocognition and brain function in individuals with or at risk for schizophrenia. Based in part on Larry’s seminal work, many in the field have come to view schizophrenia as fundamentally a disorder of dysconnection within and between certain functional networks in the brain. However, what levels or patterns of dysconnection may be sufficient for overt psychosis remains unclear. Because schizophrenia is complexly determined, clinically heterogeneous, and (frequently) chronic and debilitating, neuroimaging studies comparing those with and without this condition cannot by themselves differentiate which neural changes contribute causally, which are epiphenomena, and which are secondary to factors associated with chronicity of illness or antipsychotic drug treatment. A crucial aim is thus isolation of the changes immediately preceding the onset of psychosis that, by virtue of their temporal priority, may represent primary mechanisms in the cascades of events leading to the emergence of psychosis.

**Methods:**

Identifying such changes requires a paradigm for ascertaining at-risk individuals prior to psychosis onset and following them over time. Larry Seidman’s early work found that both patients and their first-degree relatives fail to disengage the default mode network and fail to engage task-positive networks under cognitive challenge. In the early 2000’s, in an effort to isolate changes in brain structure and function more proximal to the onset of psychosis, Larry joined with seven other investigators to launch the North American Prodrome Longitudinal Study (NAPLS). This talk will focus specifically on neuroimaging markers and on results examining baseline and longitudinal changes in brain structure and function among clinical high-risk (CHR) and control subjects, who were scanned at baseline and at 12-months or the point of conversion if it occurred earlier.

**Results:**

Converters to psychosis showed a significantly steeper rate of gray matter thinning in right superior and medial prefrontal cortex (PFC) and greater ventricular expansion than non-converters and controls. These effects were significant controlling for multiple testing and independent of exposure to antipsychotic drugs. Higher levels of proinflammatory cytokines at baseline were predictive of steeper rates of gray matter reduction in superior and medial PFC, consistent with the notion that progressive gray matter change in this context is likely to reflect dendritic retraction and synaptic pruning driven by microglial activation. This interpretation is further supported by recent evidence of genetic susceptibility mechanisms involving complement signaling in schizophrenia, variations that appear to result in over-pruning of cortical synapses in animal models. In smaller subsamples using both task-based and resting-state fMRI, CHR subjects who converted to psychosis showed a progressive decrease in global efficiency and increase in network diversity from baseline to follow-up at the point of conversion. The identified network alterations were highly correlated with each other and with progressive gray matter changes in the prefrontal cortex in converters.

**Discussion:**

These results are suggestive of a progressive loss of gray matter potentially triggered by altered immune signaling leading to over-pruning of synapses and provide preliminary evidence for longitudinal reconfiguration of resting-state and task-positive brain networks during psychosis development. The latter results appear to converge with Dr Seidman’s pioneering work on default mode and task-positive network function in individuals at genetic risk for schizophrenia.

